# Catalogue of LPS-induced transcriptional changes across vertebrates identifies syntenically conserved human long non-coding RNAs that regulate the innate immune response

**DOI:** 10.3389/fimmu.2025.1686490

**Published:** 2026-01-12

**Authors:** Marina R. Hadjicharalambous, Richard R. Ryder, Peter S. Fenwick, Louise E. Donnelly, Mark A. Lindsay

**Affiliations:** 1 Department of Life Science, University of Bath, Bath, United Kingdom; 2 National Heart and Lung Institute, Imperial College London, London, United Kingdom

**Keywords:** vertebrate, innate immunity, LPS, long non-coding RNAs, evolution, transcriptomics

## Abstract

**Background:**

Innate immunity involves the detection and removal of pathogens and is an ancient physiological response observed across all living organisms. Long non-coding RNAs (lncRNAs) are a novel family of RNA transcripts that regulate the innate immune response. Although the identification of functional lncRNAs has been hindered by their poor evolutionary sequence conservation, it has been speculated that syntenic conservation (position relative to protein coding genes) might provide an alternative approach. To examine this hypothesis, we have produced a catalogue containing the LPS-induced transcriptional changes across 27 vertebrate species, which was employed to identify syntenically conserved and functional LPS-induced human lncRNAs.

**Methods:**

Transcriptomics was employed to compare differential lncRNA expression, as well as mRNA expression, between LPS-stimulated human cells and 26 vertebrate species, including 7 primates, 10 mammals, 4 birds and 5 fish. The function of manually annotated syntenically conserved human lncRNAs was examined in LPS-stimulated monocytic THP-1 cells using antisense mediated knockdown.

**Results:**

Sequencing data from 9 LPS-stimulated human cell studies was analysed to produce a high confidence catalogue of 1036 mRNAs and 71 lncRNAs that were differentially expressed in 4 or more cell types. Examination of the mRNAs involved in the LPS signaling pathway showed evolutionary conservation across human, primates, mammals, birds and fish, one notable exception being the absence of the LPS sensing complex (MD2/TLR4/CD14) in fish. To overcome poor sequence conservation amongst lncRNAs, we employed syntenic position to show that many of the 71 lncRNAs identified in humans were conserved and induced across vertebrates, the most common being *MITA1 (IL7AS)* and *LINC02541*. Knockdown studies showed that *MITA1 (IL7AS)* and *LINC02541* negatively regulate the human LPS-induced inflammatory response in monocytic THP-1 cells. Significantly, we have identified regions/domains of *MITA1 (IL7AS)* and *LINC02541* that are conserved across vertebrates.

**Conclusion:**

This report provides a comprehensive catalogue of the transcriptional response following activation of the LPS-induced innate immune response across 9 human cell types and 26 vertebrate species. We show that many of the LPS-induced human lncRNAs are syntenically conserved and induced across vertebrate species and that this can predict a functional role in the human innate immune response.

## Introduction

Life is traditionally divided into 5 kingdoms comprised of the animals (vertebrates and non-vertebrates), plants, fungi, protista (single cell eukaryotic) and monera (bacteria and archaebacteria). The innate immune response involves the detection and removal of pathogens (i.e. non-self) and is an ancient physiological response observed in all living organisms, that is thought to have emerged at the same time as eukaryotic cells approximately 1 billion years ago (1, 2). In animals, this innate immune response is commonly mediated by specialised phagocytic cells that recognise conserved molecular structures entitled pathogen-associated molecular patterns (PAMPs) upon bacteria, viruses and fungi, through binding to pathogen recognition receptors (PPR). This recognition results in phagocytosis and induction of an inflammatory response including the release of effector molecules such as oxygen and nitrogen species, as well as widespread changes in gene expression (3). In contrast, the adaptive immune response in the form of B- and T-cell mediated IgG memory (jawed) and VLR-based memory (jawless) is only observed in vertebrate species and is thought to have emerged during the Cambrian period approximately 500 million years ago (mya) (1, 2).

Long non-coding RNAs (lncRNAs) are a novel family of RNA transcripts longer than 200 nucleotides in length and have low coding potential (4–6). Based upon their relative position to protein coding genes, lncRNAs are currently divided into three families including i) long intergenic lncRNAs (lincRNAs), which are located between protein coding genes, ii) divergent transcript (DT) lncRNAs, which share a common promoter region with a protein coding gene but are transcribed from the opposite DNA strand and, iii) antisense (AS) lncRNAs, that are transcribed across a protein coding gene but on the opposite DNA strand. LncRNAs are known to regulate a host of physiological and pathological responses, including the innate immune response (7–9). However, the identification of functional lncRNAs, as well as their mechanism of action, has been hindered by their poor evolutionary sequence conservation. This contrasts with protein coding genes where sequence conservation has permitted the identification of orthologous (and functionally comparable) genes across species. To overcome this issue, one approach has been to identify conserved lncRNAs at the transcriptional (RNA) levels based upon their syntenic position (position relative to protein coding genes) (10, 11). Using this approach, Hezroni et al. (10), analysed tissues from 17 vertebrate species and identified 1000’s of conserved lncRNAs whilst Necsulea et al. (11) identified ~ 400 lncRNAs based upon the analysis of 11 tetrapod species. This approach has also been employed to identify individual lncRNAs that show syntenic conservation between human and mouse including those that regulate the innate immune response such as *lnc13* (12), *IL7AS* (13), lnc-ulcerative colitis (*Lnc-UC*) (14), *lncRNA-GM* (15), gastric adenocarcinoma predictive linc (*GAPLINC*) (16), ncRNA inducing MHC-I and immunogenicity of tumor (*LIMIT*) (17) and *LncRNA-ISIR* (18). Significantly, Schmerer et al. have just undertaken a comprehensive analysis examining the pathway dependencies, conservation, functions and protein interaction of lncRNAs involved in the activation of innate immunity in human macrophages. Specifically, the report identifies 4 lncRNAs (*ROCKI*, *LINC00158*, *LINC01215* and *AC022816.1*) that regulate the innate immune response and show conservation across primates and mammals (19).

Activation of Toll like receptor (TLR)-4 by lipopolysaccharide (LPS), a membrane component of gram-negative bacteria, was one of the first PAMP-PRR interactions to be characterised (20). TLR4 is a member of the interleukin-1 and toll-like receptors (TIR) superfamily that forms a complex with myeloid differentiation factor 2 (MD2) and CD14, which is essential for LPS binding. Engagement of members of the TIR family stimulates a common intracellular signaling pathway culminating in the activation of transcription factors such as nuclear factor-κB (NF-κB) and activator protein-1 (AP-1) and the subsequent production of pro-inflammatory mediators (3).

Significantly, activation using LPS has become a standard approach for examining the innate immune response in vertebrates, including the differential expression of mRNAs and lncRNAs. Building on this observation, we have assembled a unique resource containing transcriptomics data from all published data on LPS-stimulated human cells, as well as LPS-stimulated cells and tissues from 26 vertebrate species (7 primates, 10 mammals, 4 birds and 5 fish). Using this catalogue, initial analysis identified 1036 mRNAs and 73 lncRNAs that were differentially expressed in 4 or more human cell types. Using these datasets, subsequent comparative transcriptomics has then been employed to characterise differential mRNA and lncRNA expression across vertebrate species and identify which LPS-induced human lncRNAs are syntenically conserved. Crucially, this analysis has shown the two most syntenically conserved and differentially expressed human lncRNAs, *MITA1 (IL7-AS)* and *LINC02541*, are negative regulators of the LPS-induced innate immune response.

## Methods

### Sourcing and indexing of the reference genomes

The fasta sequence (species.toplevel.fa.gz) of the human reference genome hg38 (human) or the relevant vertebrate genomes were sourced from Ensembl (https://ftp.ensembl.org/pub/current_fasta/). When not available from Ensembl these were sourced from either UCSC genome browser (https://hgdownload.soe.ucsc.edu/downloads.html) or NCBI (https://www.ncbi.nlm.nih.gov/datasets/genome/).

FASTA files from the relevant species were then indexed with hisat2 (21) (version 2.2.1) using the following command:

hisat2-build -p 16 <species.fa> genome

### Sourcing and alignment of RNA sequencing data

Bulk RNA sequencing data was obtained by searching the sequence read archive (SRA) using the terms lipopolysaccharide, LPS or bacteria. Data was downloaded from the SRA using fastq dump with the following command lines:

Paired-end data: fastq-dump -I –split-files <SRR file name>
Single end data: fastq-dump -I <SRR file name>

**Figure 1:**
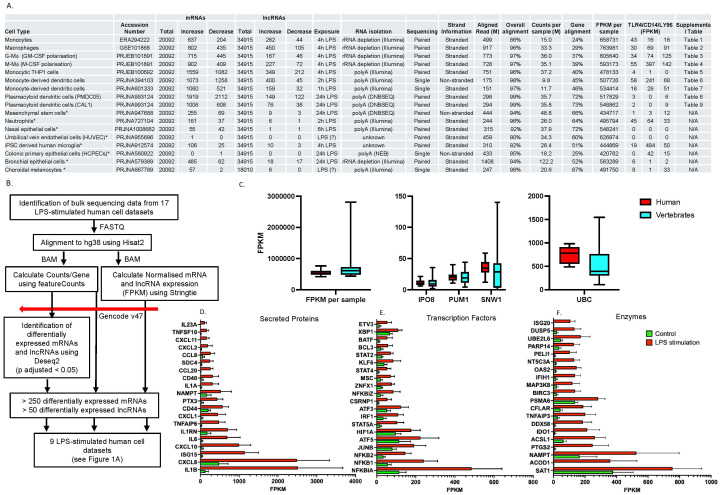
Comparative transcriptomics analysis of the LPS-induced responses in human cells. Sequencing data from 17 human cell types **(A)** was analysed using the protocol outlined **(B)** to identify differentially expressed mRNAs and lncRNAs in response to LPS-stimulation. Those datasets that showed differential expression of > 500 mRNAs and > 50 lncRNAs were deemed to have produced a robust response to LPS and employed in subsequent analysis. **(C)** shows the distribution of alignments expressed as fragments per kilobase per million mapped read (FPKM) across human and vertebrate datasets and with common housekeeping genes. Secreted proteins **(D)**, transcription factors **(E)** and enzymes **(F)** showing the largest absolute FPKM change following exposure to LPS. Data in C is a box and whisper plot showing the mean +/- SEM and the max/min value whilst data in D, E, F are the mean +/- SEM across the 9 LPS-responsive cells and all have a q < 0.005 when comparing control versus LPS stimulation (multiple paired Wilcoxon test).

**Figure 2:**
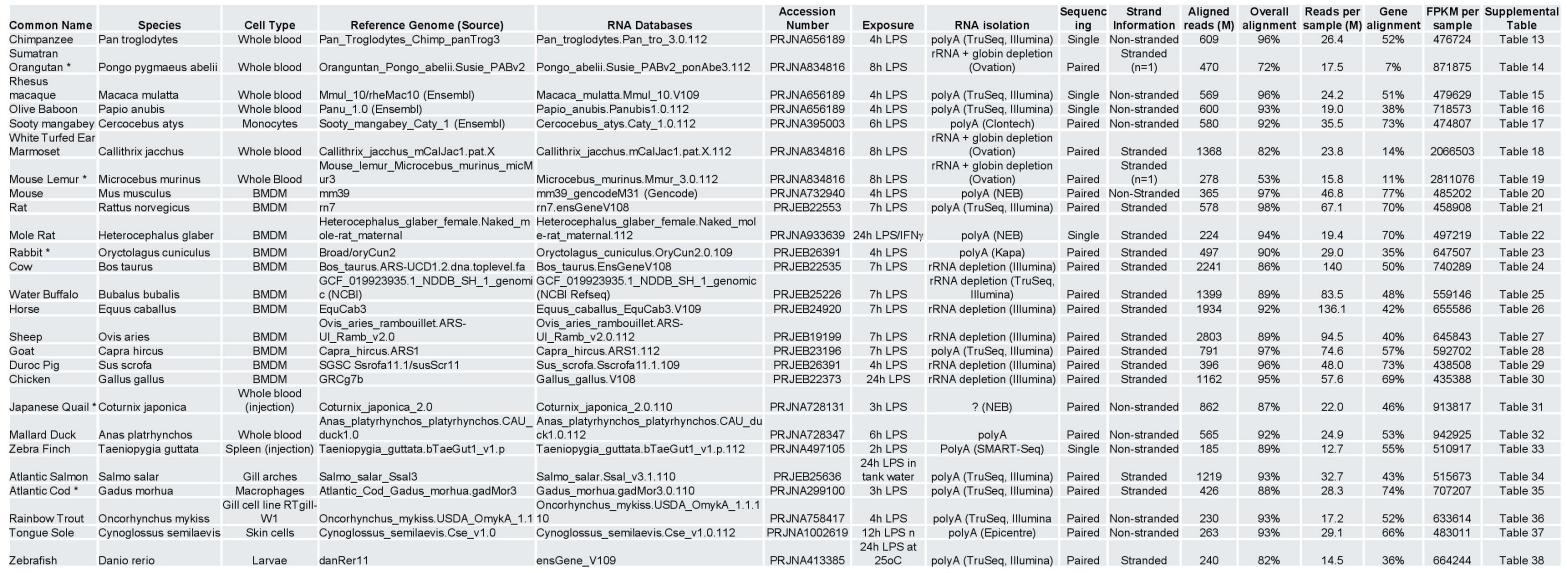
Summary of the source and analysis of LPS-induced response in 26 vertebrate species. Sequencing data from 26 vertebrate species was analysed using the protocol outlined in **Figure 1A** with the listed reference genomes and RNA databases. Unless otherwise stated in brackets, these were obtained from the Ensembl database. Table also includes the data source, period of exposure to LPS, RNA isolation technique and addition sequencing information.

Dependent upon the format of the sequencing data (**Figures 1A**, **2**), this was aligned to the relevant indexed reference genome using Hisat2 (21) (version 2.2.1) using the following command line options: Paired-end, stranded data: hisat2 –rna-strandness FR –dta -x <pathway to indexed reference genome> -1 <reverse_strand_sequence_data.fa> -2 <forward_strand_sequence_data.fa> -S <output.sam> Paired-end, non-stranded data: hisat2 –dta -x <pathway to indexed reference genome> -1 <first_strand_sequence_data.fa> -2 <forward_strand_sequence_data.fa> -S <output.sam> Single-end, stranded data: hisat2 -q –dta –rna-strandness F -x <pathway to indexed reference genome> -U <sequence_data.fa> -S <output.sam> Single-end, non-stranded data: hisat2 –dta -x <pathway to indexed reference genome> -U <sequence_data.fa> -S <output.sam>

In the case of Sumatran Orangutan, White turfed Ear Marmoset and Mouse Lemur, which did not have matching paired data, sequencing data was aligned using the following command: hisat2 –no-discordant –no-mixed –rna-strandness FR –dta <pathway to indexed reference genome> -1 <first_strand_sequence_data.fa> -2 <forward_strand_sequence_data.fa> -S <output.sam>

Output SAM files were sorted, converted to BAM files and indexed in Samtools (22) using the following command lines:

samtools sort -@ 8 –o <output.bam> <output.sam>
samtools index <output.bam>

### Gene quantification (counts/gene) and determination of differential gene expression

Gene expression (counts/gene) and differential gene expression was assessed using FeatureCounts (23) and Deseq2 (24), respectively. Alignment files (.bam) were initially converted to counts/gene in FeatureCounts using the following command line: featureCounts -p -a <RNA database.gtf> -g gene_id -o <featureCounts_output_table.txt> <Control_*.bam> <LPS_*.bam>

where Control_*.bam and LPS_*.bam are all the alignment files for a specific dataset.

The <featureCounts_output_table.txt> is then used as input for the Deseq2 Rscript programme (download from curl http://data.biostarhandbook.com/books/rnaseq/code/deseq2.r) using the following command:

cat featureCounts_output_table.txt | Rscript deseq2.r AxB > results_deseq2.csv

where A and B are the n numbers for the control and LPS repeats.

### Determination of normalised gene expression

Normalised gene expression from each alignment file (.bam), which accounts for both gene length and the total number of reads, is given as FPKM (fragments per kiobase of transcript per million reads) and was determined using Stringtie2 (25) using the following command line: stringtie <output.bam> -G <RNA database.gtf> -e –A <gene_quantification_table.txt>

### *Ab initio* assembly and the identification of syntenic lncRNAs

The alignment files for LPS samples were merged and indexed using Bamtools (26) and Samtools (22) to produce a single file for each experiment using the following command line:

bamtools merge -in <LPS_1.bam> -in <LPS_X.bam> -out <Non_sorted_merged.bam> samtools sort -@ 4 -o <Merged.bam> <Non_sorted_merged.bam>
bamtools index -in <Merged.bam>

We then performed *ad intio* transcript assembly with Stringtie2 (25) using the following command line:

Stringtie <Merged.bam> -o <Merged.gtf>

Normalised gene expression, gene quantification (counts/gene) and differential gene expression was then determined using the <Merged.gtf>, as described previously.

### Identification of species orthologs of human genes

We extracted the human orthologous genes for a particular species from Ensembl 112. Where there were multiple orthologs against a specific human gene, these were sorted to produce one ortholog using the following criteria:

%id. query gene identical to target [Species] gene (high to low)
Gene-order conservation score (high to low)
Orthology confidence [0 low, 1 high]
P adjusted FPKM (low to high)
Absolute expression FPKM (high to low)

### Multiple sequence alignment and identification of conserved domains

Strand specific sequences for syntenic version of *MITA1 (IL7AS*) (**Supplementary Data 1**) and *LINC02541* (**Supplementary Data 2**) were extracted from the relevant reference genomes (**Figure 2**) using the relevant STRG code (**Supplementary Table S40**). These FASTA file were then submitted for multi-sequence alignment using multiple alignment using fourier transform (MAFFT - www.ebi.ac.uk/jdispatcher/msa/mafft?stype=dna) or identification of domains (up to 30 nucleotides) using the XSTREME in meme suite (https://meme-suite.org/meme/), with comparison against *MITA1 (IL7AS)* and *LINC02541*.

### Culture and LPS-stimulation of human monocytic THP-1 cells

Monocytic THP-1 cells were cultured in growth media (RPMI supplemented with 10% (v:v) FCS, 2 mM L-glutamine, 100 U/ml penicillin, 100 μg/ml streptomycin and 50 nM of 2-Mercaptoethanol (all GIBCO, Life Technologies) and incubated in a 37°C, 5% (v:v) CO2 humidified incubator. Cells were stimulated for 4h with/without 1 μg/ml LPS (E. coli 055:B5, Sigma Aldrich). Total RNA was extracted using the RNeasy kit (Qiagen), which included an on-column DNase treatment (Qiagen), according to the manufacturer’s guidelines. RNA concentration was determined using the Qubit 2.0 (Life Technologies). RNA quality was measured using the Agilent Bioanalyser and produced RIN values of >8.0. Samples were dispatched to BGI Genomics (Hong Kong, China) where they were processed using the Illumina TruSeq (polyA) library kits prior to 100 nucleotide paired-end, stranded sequencing. Data is deposited in the ENA under PRJEB100692 and has been included as part of the analysis described in **Figure 1**.

### Investigation of lncRNA function in human monocytic THP-1 cells

Human monocytic THP-1 cells were transfected with antisense locked nucleic acid (LNA) GapmeRs against IL7AS at a final concentration of 30 nM in 100 μl of serum- and antibiotic-free RPMI-1640 medium, supplemented with 5μl of HiPerFect (Qiagen). The LNA GapmeRs mix was added to each well of a 24-well plate and THP1 cells (5x10^5^ cells per well) in 100 μl of their growth complete medium were added on top of the LNA GapmeRs mix. Cells were then incubated for 16h. The following day cells were diluted with 400 μl of complete medium and stimulated for 4h with/without 1 μg/ml LPS (E. coli 055:B5, Sigma Aldrich). Total RNA was extracted using the RNeasy kit (Qiagen), which included an on-column DNase treatment (Qiagen), according to the manufacturer’s guidelines. RNA concentration was determined using the Qubit 2.0 (Life Technologies). RNA quality was measured using the Agilent Bioanalyser and produced RIN values of >8.0. Samples were dispatched to BGI Genomics (Hong Kong, China) where they underwent polyA isolation prior to 100 nucleotide paired-end, stranded sequencing. Data is deposited in the European Nucleotide Archive (ENA) under PRJEB101967.

### Human monocyte-derived macrophages

Monocyte derived macrophages (MDMs) were generated from monocytes isolated from peripheral blood mononuclear cells using a Percoll gradient and adherence technique, followed by culture for 12 days in RPMI supplemented with 10% v/v fetal calf serum 2 mM L-glutamine and 100 µg/ml penicillin/streptomycin with either 2 ng·mL^−1^ GM-CSF (cat. no. 7954-GM-059/CF, Bio-Techne, Abingdon, UK) to generate G-Mφ or 100 ng·mL^−1^ M-CSF (cat. no. 216-MC-500, Bio-Techne) to generate M-Mφ. MDMs were then stimulated for 4h with/without 1 μg/ml LPS (E. coli 055:B5, Sigma Aldrich). Total RNA was extracted using the RNeasy kit (Qiagen), which included an on-column DNase treatment (Qiagen), according to the manufacturer’s guidelines. RNA concentration was determined using the Qubit 2.0 (Life Technologies). RNA quality was measured using the Agilent Bioanalyser and produced RIN values of >8.0. Samples were dispatched to BGI Genomics (Hong Kong, China) where they underwent Ribozero isolation prior to 100 nucleotide paired-end, stranded sequencing. These studies were performed under ethic approval number 09/H0801/85 from the National Research Ethics Service (NRES), South West London REC1. Data is deposited in the European Nucleotide Archive (ENA) under PRJEB101891 and has been included as part of the analysis described in **Figure 1**.

### Availability of data and materials

All sequencing data is available through NCBI sequence read archive (SRA) or European Nucleotide Archive (ENA) using the accession numbers listed in the Figures or Methods.

All **Supplementary Data** can be obtained at FigShare at the following DOI: 10.6084/m9.figshare.30868874.

## Results

### Catalogue of differential mRNA and lncRNA expression following LPS-induced activation of human innate immune response

To produce a comprehensive catalogue of high confidence, differentially expressed mRNAs and lncRNAs following activation of the human innate immune response, we identified bulk RNA sequencing data from 17 human primary cells or cells lines, following stimulation with LPS for up to 24h (**Figure 1A**). Differentially gene expression including mRNAs and lncRNAs, was assessed with the GENCODE database (v47), based on a p-adjusted value < 0.05 and an absolute change of > 1 FPKM. Of relevance, Gencode v47 includes updated annotations based on long read data which has resulted in a substantial increase in annotated lncRNAs from 20310 (v46) to 34915 (v47), and a smaller increase in mRNAs from 19411 (v46) to 20092 (v47) (27).

Comparison of alignment statistics across these datasets (mean ± SD) demonstrated that whilst overall alignment was high (95 ± 3%), there was large variation in the aligned reads (496 ± 327M), counts per sample (34 ± 25M) and % alignment to known genes (51 ± 17). In contrast, normalisation of the data using Fragments per Kilobase per Million mapped fragment (FPKM) produce a much smaller variation across samples (543 ± 88M) and indicated that this metric might be employed to compare gene expression across datasets. In light of this observation, we examined the FPKM of 39 previously identified housekeeping genes (28–31) and showed that ubiquitin C (UBC) (SD = 24%), importin 8 (IPO8) (SD = 37%), pumilio RNA binding family member 1 (PUM1) (SD = 35%) and SNW domain containing 1 (SNW1) (SD = 35%) demonstrated the least variation in expression across human datasets (**Figure 1C**) (**Supplementary Table S10**).

To ensure that we analysed cells that gave a robust LPS response, we excluded those datasets with < 500 differentially expressed mRNAs and < 50 differentially expressed lncRNAs, leaving 9 datasets for downstream analysis (**Figure 1A**, **Supplementary Data 10**). Interestingly, cell responses to LPS did not appear to be linked to the expression levels of TLR4, CD14 or LY96, the components of the complex responsible for binding LPS (**Figure 1A**).

Compilation of data across these 9 cell types showed differential expression of 7112 mRNAs in a least one cell type (**Supplementary Data 10**). To reduce this number for subsequent comparison across vertebrate species, we focused on those genes that were differentially expressed in 4 or more cell types. This identified 1036 differentially expressed mRNAs, of which the majority were up-regulated (746 mRNAs). (**Supplementary Table S11**). As might be expected, KEGG analysis of the up-regulated genes detected multiple pathways associated with immunity/inflammation, the most significant being TNF-signaling (5.4 x 10^-21^), NOD-like signaling (6.9 x 10^-17^) and NF-κB signaling (8.8 x 10^-17^) but also necroptosis (2.4 x 10^-6^) and apoptosis (2.3 x 10^-4^). This indicates that LPS induces both an inflammatory and apoptotic response. No pathways were identified for the down-regulated genes. To classify these up-regulated genes, we cross-referenced our gene list with data from the human protein atlas (https://www.proteinatlas.org/) (32) and identified 72 secreted proteins, 127 transcription factors, 229 enzymes, 67 transporters, 19 G-protein coupled receptors and 4 voltage-gated ion channels (**Supplementary Table S11**). Examination of the mRNAs showing the largest absolute change (FPKM) indicated that IL1β and CXCL8 are highest amongst secreted proteins (**Figure 1D**), which also includes additional cytokines (IL1a, IL6 and IL23A) and chemokines (CXCL1/2/10/11). Amongst the transcription factors (**Figure 1E**), there was upregulation of mRNAs coding for NF-κB (NFKBIA, NFKB1, NFKB2, NFKBIZ, BCL3), STAT (STAT4, STAT5A and STAT2) and AP-1 (JUNB, ATF5, ATF3) signaling complexes. The most expressed enzymes included those involved in metabolism (SAT1, ACOD1, NAMPT, ACSL1, IDO1), as well as the well-known inflammatory enzyme, PTGS2 (COX2) which catalyses the production of prostaglandins (**Figure 1F**).

In the case of lncRNAs, we observed 979 lncRNAs that were differentially expressed in a least one cell type. As with mRNAs, we focused upon lncRNAs that were differentially expressed in 4 or more cells, which identified 96. These lncRNAs were manually annotated to remove any that might represent mRNAs and classified as either lincRNAs (located between protein coding genes), antisense (transcribed across a protein coding gene but on the opposite DNA strand) or divergent transcripts (which share a common promoter region with a protein coding gene but are transcribed from the opposite DNA strand). This analysis ultimately identified 73 lncRNAs that were differentially expressed in response to LPS (67 up-regulated and 6 down-regulated) including 32 lincRNAs, 18 antisense (AS) and 23 divergent transcripts (DT) (**Supplementary Table S12**). Amongst the lincRNAs were 5 miRNA host genes (MIR155HG, MIR3945, MIR3142, MIR122HG, MIR9-1HG), that code for miR-155, miR-3945, miR-146a, miR-222 and miR-9-1, as well as 2 small nucleolar RNA host genes (SHNG12 and SHNG15).

### Catalogue of differentially expressed mRNAs and lncRNAs following LPS-induced activation of innate immune response across vertebrates

To examine differential expression of mRNAs and lncRNAs across vertebrates, we identified data from 26 species including 7 primates [chimpanzee (33), orangutan (34), macaque (33), baboon (33), sooty mangabey (35), marmoset (34) and mouse lemur (34)], 10 mammals [mouse (36), rat (37), mole rat (38), rabbit (39), cow (37), water buffalo (37), horse (37), sheep (39), goat (37) and pig (37)], 4 birds [chicken (40), quail (41), duck (42) and zebra finch (43)] and 5 fish [salmon (44), cod (45), trout (46), sole (47) and zebrafish (48)] (**Figure 2**). The differential expression of mRNAs and lncRNAs across these vertebrate species (**Supplementary Tables S13-S38**) was analysed using gene annotations provided by Ensembl, following alignment to the relevant reference genomes (**Supplementary Tables S13-S38**). Exceptions to this approach included mouse data, which was annotated using Gencode M31 and water buffalo data, which were annotated using data from NCBI Refseq (**Figure 2**).

In contrast to the human data which were obtained from isolated cells, the vertebrate datasets was derived from a variety of sources including bone marrow-derived macrophages (BMDM) in mammals and chickens, as well as whole blood in primates and birds. Other differences included the duration of LPS exposure, which varied from 2 to 24 h, as well as the RNA isolation techniques employed. As with humans, comparison across these datasets demonstrated good overall alignment (92 ± 4%) but a large variation in the total aligned reads (802 ± 670M), reads per sample (44 ± 36M) and % alignment to known genes (50 ± 14%). In contrast to human cells, FPKM demonstrated wide variation (75 ± 53M) although this appeared to be caused by data derived from a single publication that employed whole blood with rRNA and globulin depletion. In the absence of these samples, FPKM variation was 60 ± 14M (**Figure 1C**). Once again these observations suggested that FPKM could be employed to compare gene expression across samples. Examination of housekeeping genes identified in human cells, showed that IPO8 and PUM1 (but not SNW1 and UBC) were also conserved across vertebrates (**Figure 1C**).

**Figure 3:**
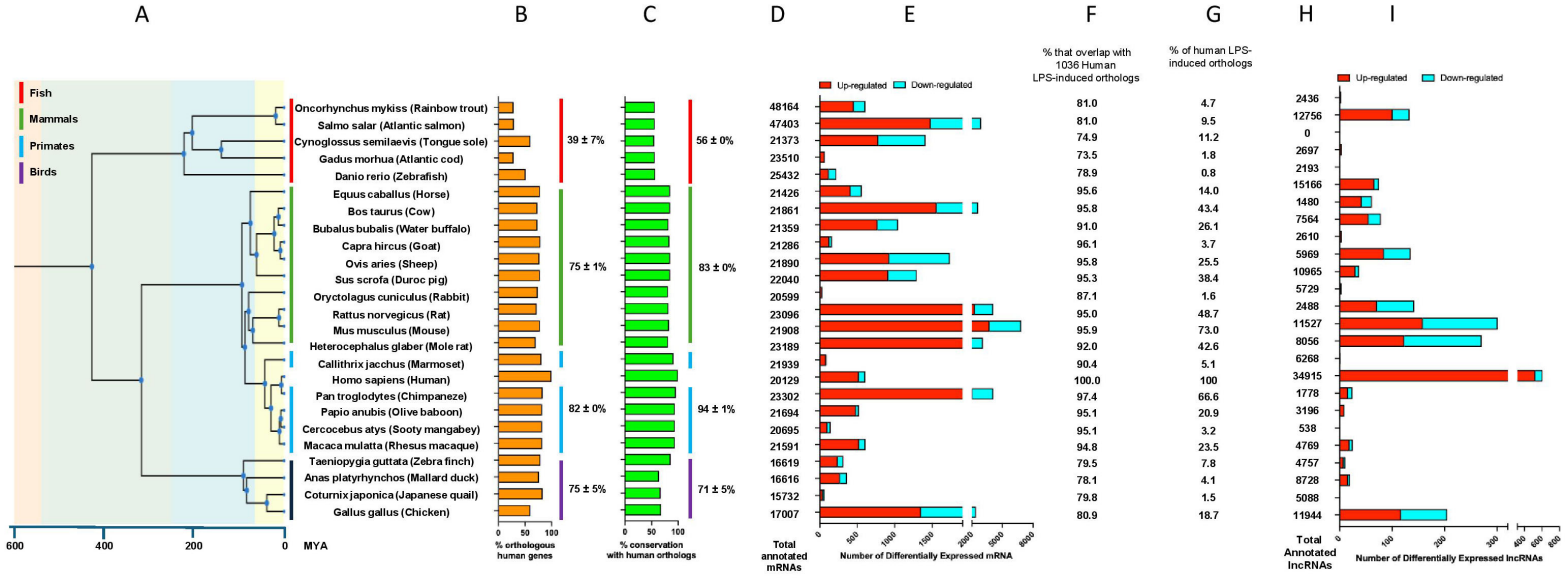
LPS induced differential expression of mRNAs and lncRNAs across 26 vertebrate species. **(A)** shows the evolutionary branching of the vertebrates species examined in this report, **(B)** shows the % of mRNAs in the stated species that have human orthologs, **(C)** shows the % conservation in the species mRNAs with the human mRNA orthologs, **(D)** shows the number of annotated mRNAs in the indicated species, **(E)** shows the number of differentially expressed mRNAs following LPS stimulation, **(F)** shows the % of mRNAs orthologs in the indicated species that match the 1016 mRNAs that are differentially expressed in humans following LPS stimulation, **(G)** shows the % of mRNAs in the indicated species that are differentially expression (column E) and that overlap with the 1016 mRNAs that are differentially expressed in human following LPS stimulation, **(H)** shows the number of annotated lncRNAs in the indicated species and **(I)** shown the number of differentially expressed lncRNAs following LPS stimulation.

**Figure 3A** shows the evolutionary branching of the vertebrate species employed in this report including (jawed) fish (emerged ~420 mya), birds (emerged ~160 mya), mammals (emerged ~225 mya), primates (~85 mya) and humans (~0.3 mya). From **Figure 3D**, it can be seen that the mean number of mRNAs in primates, mammals, birds and fish are 21147, 21865, 16493 and 23585 (when factoring in the whole genome duplication in salmon and trout), respectively. This is comparable to the ~20,000 in human (GENCODE v47). Likely due to variation in availability/depth of sequencing data and annotation, the numbers of lncRNAs varies from 538 (Sooty Mangabey) to 15166 (horse), which are all considerably lower than the 34915 annotated in humans (GENCODE v47) (**Figure 3H**). Examination of the LPS-induced transcriptional response across vertebrates (**Figure 3E**) showed considerable differences in the number of differentially expressed mRNAs which ranged from 36 mRNAs in rabbits to 6852 mRNAs in mice. We observed similar variability in the number of differentially expressed lncRNAs that ranged from 0 to 301 (mouse), compared with the 607 in human (**Figure 3I**). In the case of orangutans and mouse lemurs, we had insufficient replicates (i.e. n=1) to be able to perform statistical analysis. In the subsequent evaluation, data were grouped into primates, mammals, birds and fish.

### LPS signaling pathways were conserved across vertebrates

**Figure 4:**
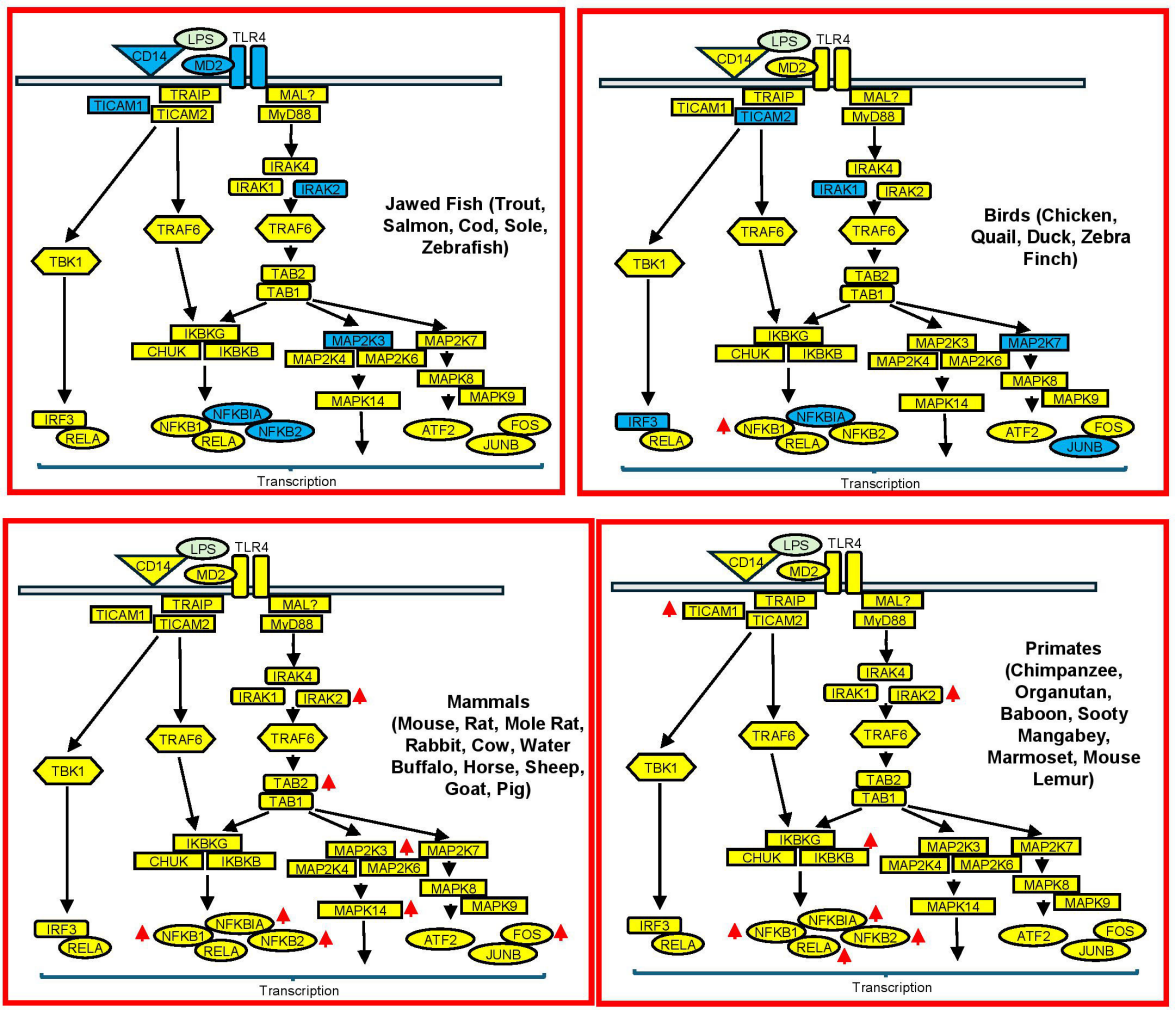
Comparison of the LPS signaling pathways in vertebrates. Transcriptomics data from LPS-stimulated cells and tissues of 26 vertebrates including 7 primates, 10 mammals, 4 birds and 5 fish were employed to examine the expression of the proteins involved in the LPS signaling pathway. Protein expressed at > 1 FPKM across > 50% of cells/tissues are shown in yellow whilst those proteins that were significantly differentially expression are indicated with a red arrow.

We next investigated whether the LPS signaling pathway is conserved across vertebrates. As shown in **Figure 4**, primates and mammals express all the proteins (shown in yellow) seen in humans; many of which are upregulated in response to LPS, particularly IRAK2 and members of the NF-κB transcription complex. Similarly, despite the emergence of birds and jawed fish approximately 180 and 420 million years ago, these vertebrates still express the majority of the proteins in the LPS signaling pathway. The notable exception is that fish lack the components of the LPS receptor, including CD14, TLR4 and MD2, which means that they must detect and respond to LPS via an alternative pathway. Overall, this indicates that the LPS signaling pathway is highly conserved across vertebrates confirming that this pathway emerged early in animal evolution.

### Comparison of the LPS induced mRNA response across vertebrates

To allow comparison with the human LPS response, we initially employed Ensembl to identify the human orthologs of the various vertebrate mRNAs (**Supplementary Table S39**). Reflecting their evolutionary divergence, the mean number of human mRNAs orthologs in primates, mammals, birds and fish were 82%, 75%, 75% and 39% (**Figure 3B**), with mean % sequence conservation across these orthologous mRNAs being 94%, 83%, 71% and 56%, respectively (**Figure 3C**).

**Figure 5:**
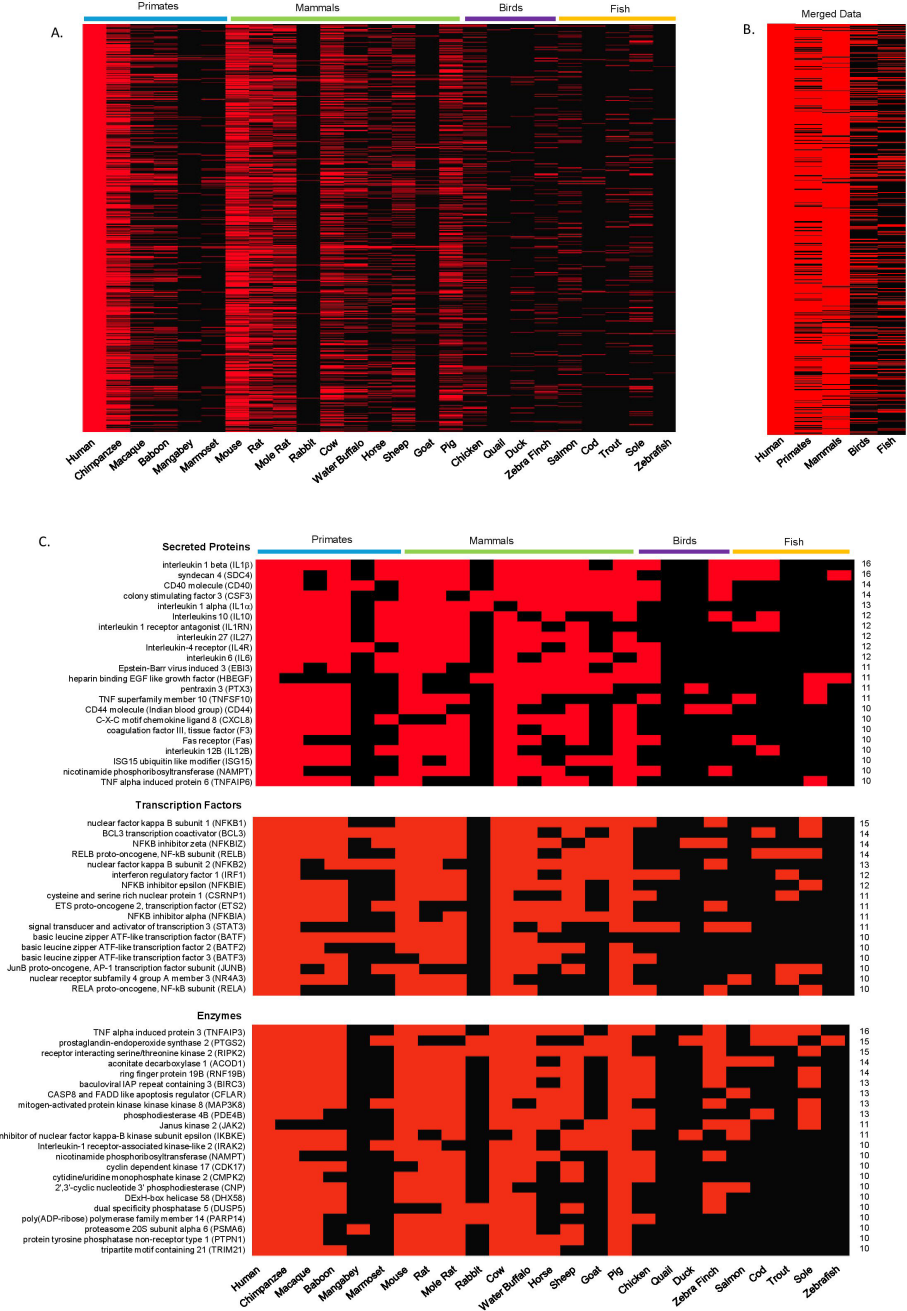
Comparison of the LPS induced transcription response in humans with that in vertebrates. Transcriptomics data from LPS-stimulated cells and tissues of 26 vertebrates including 7 primates, 10 mammals, 4 birds and 5 fish were employed to identify which orthologs match those observed in LPS-stimulated human cells (1036 mRNAs). **(A)** heatmap comparing individual species against human LPS-induced mRNAs, **(B)** heatmap comparing merged group data from primates, mammals, birds and fish against human LPS-induced mRNAs, **(C)** block diagram showing the most commonly expressed (conserved) secrete proteins, transcription factors and enzymes. A redline or block indicates that the specific ortholog is differentially expressed in the indicated species or group (p-adjusted < 0.05).

Our previous analysis of the LPS-induced response in human cells identified 746 up-regulated and 290 down-regulated mRNAs (**Supplementary Table S11**). The data in **Figure 3F** and heatmap in **Figure 5A** shows the orthologs of these 1036 human mRNAs that are also differentially expressed across the various vertebrate species. Following exclusion of those datasets that did not give a robust response (< 250 differential expressed mRNAs), the mean number of human LPS-induced ortholog genes that were also seen in primates, mammals, birds and fish was 37 ± 15%, 39 ± 6%, 9 ± 5% and 8 ± 2%, respectively (**Figure 3G**). Interestingly, following the merger of the data within each group, it was shown that the majority of LPS induced mRNAs in humans are also seen primates (70%) and mammals (92%), although this number is dramatically reduced in birds (27%) and fish (23%) (**Figure 5B**). Using this data, it was shown that 70 mRNAs are differential expressed across humans, primates, mammals, birds and fish (**Supplementary Table S11**).

As with the human data (**Figures 2D–F**), we classified these orthologous mRNAs by function into secreted proteins, transcription factors and enzymes, and plotted those expressed in the highest number of vertebrates (**Figure 5C**). Among secreted proteins, the inflammatory mediator IL1β and transmembrane proteoglycan, syndecan 4 (SDC4), were induced in the largest number of vertebrates (16/24) and across both primates, mammals, birds and fish. Interleukin-10 (IL10), interleukin-1 receptor antagonist (IL1RN), penetraxin (PTX), TNF superfamily member 10 (TNFSF10), heparin binding EGF like growth factor (HBEGF) and Fas receptor (Fas) were also induced across all 4 vertebrate groups. Interestingly, a number of common inflammatory mediators such as IL1a, IL27, CXCL8 and IL12B were only induced in primates and mammals. As has already been highlighted, the most often induced transcription factors were members of the NF-κB complex (NFKB1, RELB, NFKB2, NFKBIZ, NFKBIE, NFKBIA, REL). In addition, we observed induction of IRF1 and STAT3 across all 4 groups. The most conserved and induced enzymes included kinases (RIPK2, MAP3K8, IRAK2, CDK17), and others that have been implicated in the regulation of inflammation including PTGS2 (COX2) and PDE4B. The most conserved enzyme was TNF alpha induced protein 3 (TNFAIP3) (16/24), RIPK (15/24) and PTGS2 (15/24), which along with RNF19B, ACOD1, BIRC2, CFLAR, PDE4B and CNP, were induced in both primates, mammals, birds and fish (**Figure 5C**).

### Identification of syntenically conserved LPS-induced lncRNAs across vertebrates

**Figure 6:**
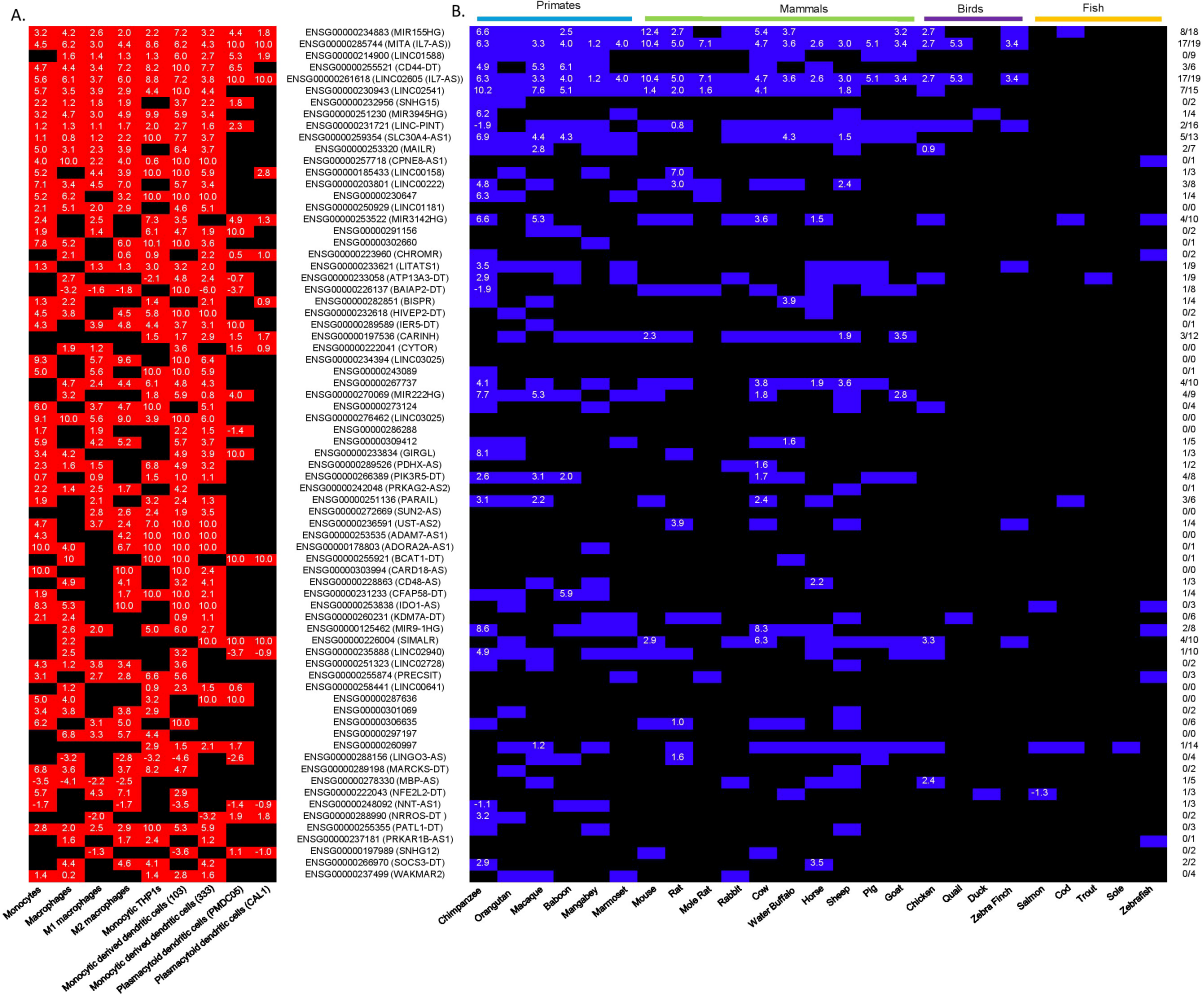
Expression and LPS-induced increases in lncRNAs across vertebrates. **(A)** Red boxes indicate which lncRNAs were significantly increased (+) or decreased (–) (p-adjusted < 0.05) in the relevant human cell type in response to LPS stimulation whilst the number in white gives the log2 fold change and, **(B)** Blue boxes indicated which vertebrate species demonstrated the presence of a syntenic lncRNA. The presence of a white number indicates a significant (p-adjusted < 0.05) increase (+) or decrease (-) in expression whilst the magnitude of the number is the log2 fold change. in the relevant vertebrate species.

We had previously identified 73 lncRNAs that were differentially expressed in 4 or more human cell types in response to LPS exposure (**Figure 6A**, **Supplementary Table S12**). To determine whether these LPS-induced lncRNAs are syntenically conserved across vertebrates, we undertook *ab initio* transcript assembly using the RNA sequencing data from the 26 vertebrate species and employed syntenic position to identify potential orthologs (**Figure 6B**, **Supplementary Tables S13A-S38A**). These *ab initio* transcript assemblies were also employed to determine differential expression and the log2 fold change (**Figure 6B**, **Supplementary Table S40**). Using expression in a least 2 species as a threshold, we showed that of the 73 lncRNAs identified in humans, 39 lncRNAs were expressed in primates, 32 lncRNAs in mammals, 5 lncRNAs in birds and a 4 lncRNAs in fish (**Figure 6B**, **Supplementary Table S40**). Based on significant differential expression we showed that 11 lncRNAs were induced in primates (*MIR155-HG*, *MITA1*, *CD44-DT*, *LINC02605*, *LINC02541*, *SLC30A4-AS*, *MIR3142HG*, *MIR222HG*, *PIK3R5-DT*, *PARAIL*, *MIR9-1HG*), 11 in mammals (*MIR-155-HG*, *MITA1*, *LINC02605*, *LINC02541*, *SLC30A4-AS*, *LINC00222*, *CARINH*, *ENSG00000267737*, *ENSG00000270069*, *PIK3R5-DT*, *MIR9-1HG*), 2 in birds (*MITA1* and *LINC02605*) and none in fish (**Figure 6B**). Using expression alone, the most commonly expressed lncRNAs across the 25 species were *MIR-155-HG* (18/25), *MITA1* (18/25), *LINC02605* (18/25), *LINC02541* (15/25) and *LINC-PINT* (16/25) (**Figure 6B**). Based on differential expression (**Supplementary Tables S40**), *MITA1*, *LINC02605*, *LINC02541* and *MIR-155-HG* were shown to demonstrate the most consistent induction being significantly up-regulated in 17/19, 17/19, 7/15 and 8/18 species, respectively (**Figure 6B**). Other syntenic lncRNAs that were differentially expressed across multiple species included *SLC30A4-AS* (5/13), *MIR3142-HG* (4/10), *ENSG00000267737* (4/10) and *SIMALR* (4/10). Of relevance, *MIR155HG* and *MIR3142*, which are the host genes for miR-155 and miR-146a, have previously been shown to be master regulators of innate and adaptive immune response (7, 9). This indicates that our analysis pipeline provides a robust approach to the identification of functionally relevant lncRNAs.

### *MITA1 (IL7AS)* and *LINC02541* are syntenically conserved and differentially expressed across vertebrates

We have shown that *MIR155HG*, *MITA1*, *LINC02605* and *LINC02541* were syntenically conserved and differentially expressed in human, primate, mammals and/or birds in response to LPS stimulation. Since the action of *MIR155HG* is likely mediated through the production of miR-155, a well-known regulator of the innate immune response, we focused on *MITA1*, *LINC02605* and *LINC02541*. Significantly, we have previously shown that *MITA1* and *LINC02605* are isoforms of a lncRNA located antisense to IL7, which we had previously named *IL7-AS*. Given this previous observation we shall forthwith refer to *MITA1* and *LINC02605*, as *MITA1 (IL7AS)*.

**Figure 7:**
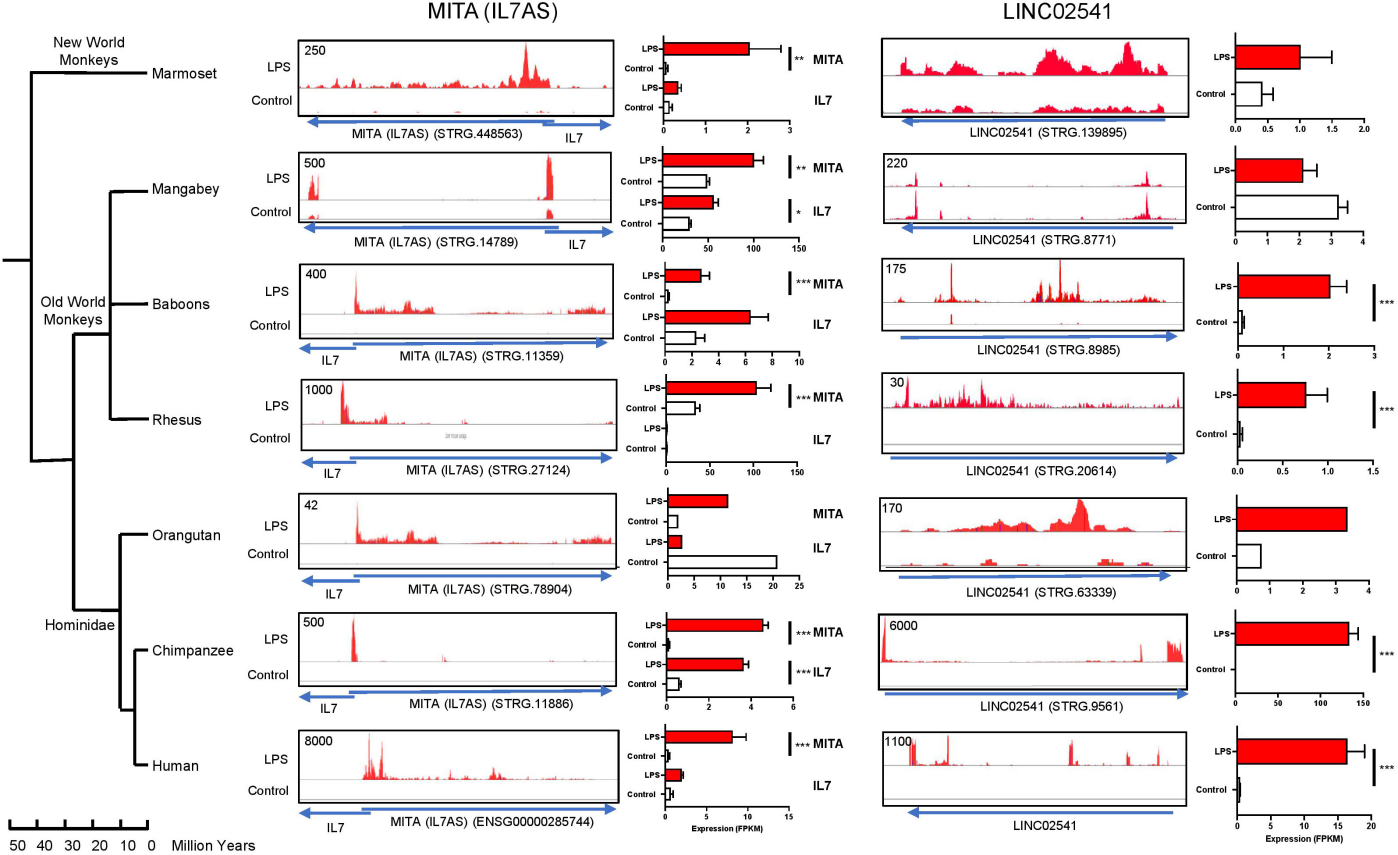
LPS-induced *MITA1 (IL7AS)* and *LINC02541* expression in primates. Following *ab initio* assembly using the RNA sequencing data from the indicated primates, the syntenic location was employed to identity and quantify the relevant orthologs for *MITA1 (IL7AS)* and *LINC02541* (STRG number derived from **Supplementary Tables S13A-S38A**). Histograms of the control and LPS alignment data are displayed using the Integrative Genomics Viewer (IGV). Data on *IL7* expression were derived from **Supplementary Tables S13-S38**. Expression analysis for *IL7*, *MITA1 (IL7AS)* and *LINC002541* are based on FPKM and are mean +/- SEM of 3–10 experiments where *p < 0.05, **p < 0.01 and ***p < 0.001 (unpaired T-test).

**Figure 8:**
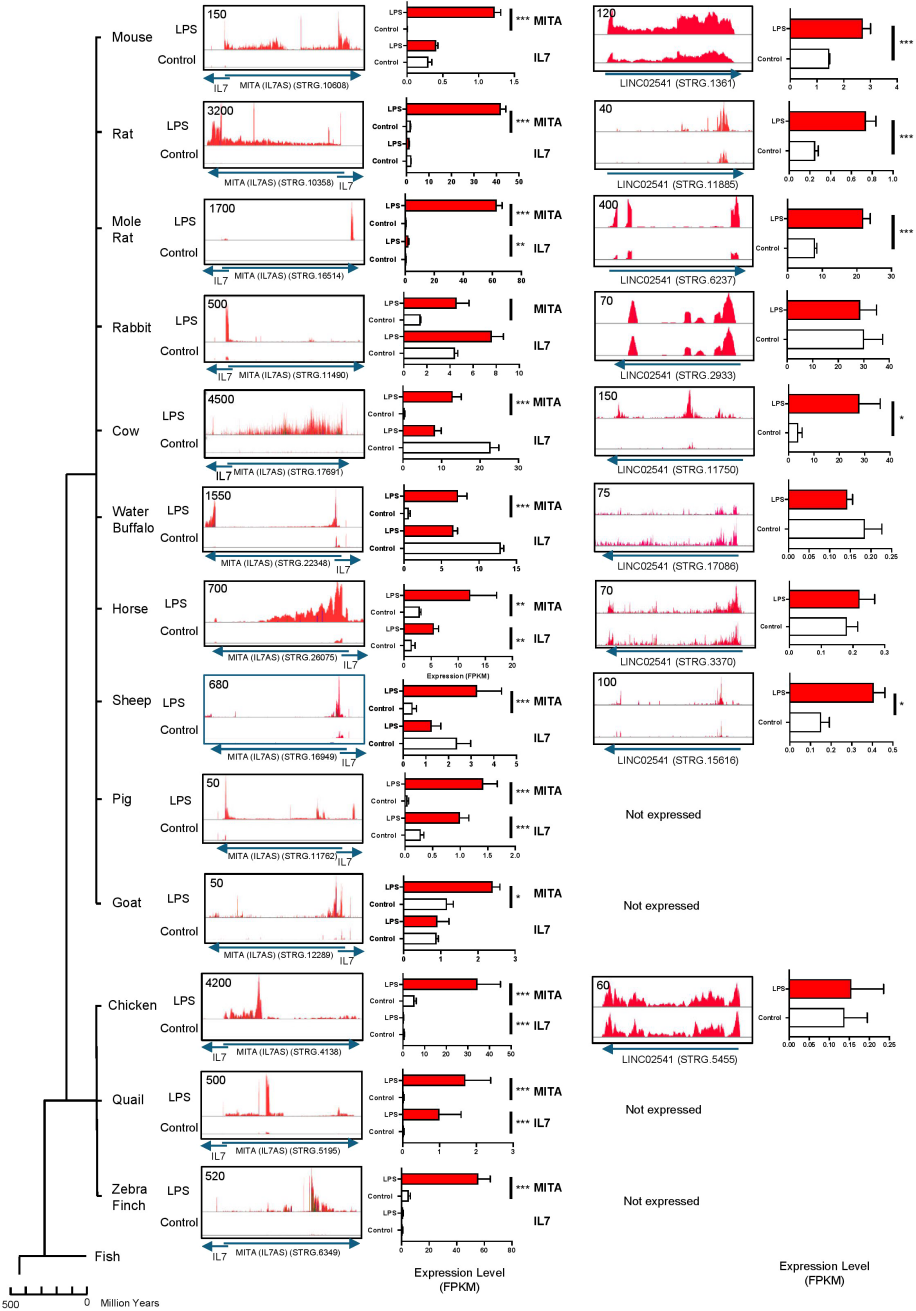
LPS-induced *MITA1 (IL7AS)* and *LINC02541* expression in mammals and birds. Following *ab initio* assembly using the RNA sequencing data from the indicated mammals and birds, the syntenic location was employed to identity and quantify the relevant orthologs for *MITA1 (IL7AS)* and *LINC02541* (STRG number derived from **Supplementary Tables S13A-S38A**). Histograms of the control and LPS alignment data are displayed using the Integrative Genomics Viewer (IGV). Data on *IL7* expression were derived from **Supplementary Tables S13-S38**. Expression analysis for *IL7*, *MITA (IL7AS)* and *LINC002541* are based on FPKM and are mean +/- SEM of 3–10 experiments where *p < 0.05, **p < 0.01 and ***p < 0.001 (unpaired T-test).

*Ab initio* transcript assembly using data obtained from LPS-stimulated human macrophages showed that these lncRNAs consisted of multiple transcripts. Thus, *MITA1 (IL7AS)* contained > 9 potential exons that could be assembled into 11 transcripts (**Supplementary Figure S1A**) whilst *LINC02541* contained > 13 potential exons that could be assembled into 19 transcripts (**Supplementary Figure S1B**). To confirm the differential expression data, we visually examined the syntenic versions of these lncRNAs (STRG numbers) across primates (**Figure 7**), as well as mammals and birds (**Figure 8**) using the relevant alignment files within the IGV genome browser. This analysis confirmed LPS-induced *MITA1 (IL7AS)* expression in all primates, mammals and bird species although this was not significant in orangutans and rabbits. Given that fish do not express IL7, we were unable to determine *MITA1 (IL7AS)* expression using the data from salmon, cod, trout, sole and zebrafish (data not shown). To ascertain whether the increase in *MITA1 (IL7AS)* might be linked to that of IL7, we also assessed the expression of the latter (**Figures 7**, **8**). However, this appeared unlikely since only 7 of 19 species demonstrated a significant increase in IL7 following exposure to LPS and, furthermore, the magnitude of the *MITA (IL7AS)* (FPKM: 17.5 +/- 5.4) response was significantly elevated (p = 0.004, paired t-test) compared with IL7 (FPKM: 0.5 +/- 2.0). Although not as prevalent, we observed significant induction in the syntenic versions of *LINC02541* in primates (chimpanzee, rhesus, baboons) and mammals (mouse, rat, mole rat, cow, sheep) but not in birds or fish (**Figures 7**, **8**).

In summary, we have shown that human LPS-induced *MITA (IL7AS)* and *LINC02541* are syntenically conserved across vertebrate species and are also induced in response to LPS exposure. Subsequent studies investigated whether these LPS-responsive human lncRNAs contained conserved regions and examined their functional role in the innate immune response.

### *MITA1 (IL7AS)* and *LINC02541* have short regions that are conserved across vertebrate species

**Figure 9:**
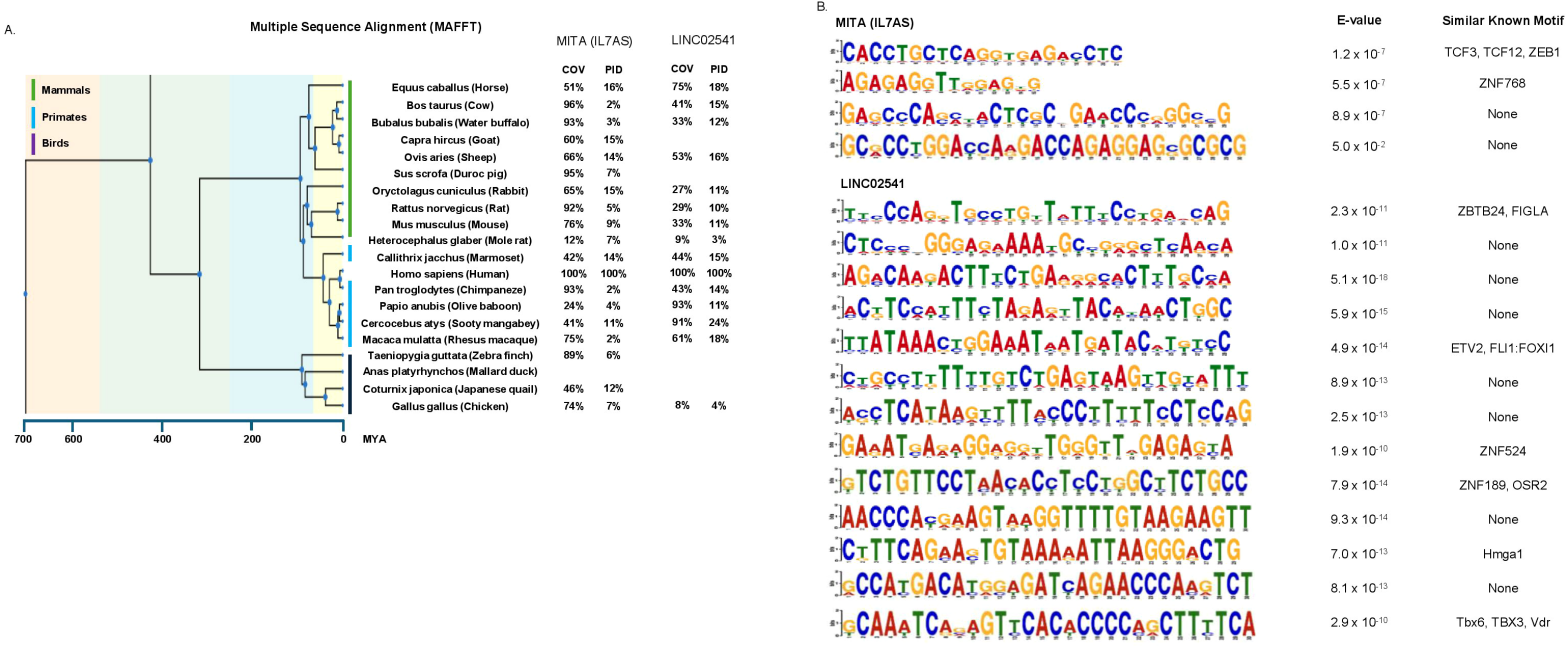
Identification of conserved regions in *MITA1 (IL7AS)* and *LINC02541* across vertebrate species. The sequences of syntenic versions of *MITA1 (IL7AS)* (**Supplementary Data 1**) and *LINC02541* (**Supplementary Data 2**) were extracted from the relevant reference genome. These sequences were then compared against *MITA1 (IL7AS)* or *LINC02541* by **(A)** multiple sequence alignment (MAFFT) to assess coverage (COV) and percentage identity (PID) or **(B)** detection of motifs/domains (MEME suite).

Having established that the syntenic version of *MITA1 (IL7AS)* and *LINC02541* are induced in response to LPS stimulation across vertebrate species, we proceeded to examine whether there are conserved regions (**Figure 9A**) and domains/motifs (**Figure 9B**). Multiple sequence alignment of the vertebrate species against *MITA1 (IL7AS)* showed an average coverage (COV – the % of the vertebrate species that is covered by *MITA1*) of 66% and average percentage identify (PID – proportion of positions in the vertebrate species that has a pairwise alignment with *MITA1 (IL7AS)* that is the same) of 8%. This did not differ significantly between primates (COV - 55%; PID – 7%), mammals (COV - 71%: PID – 9%) and birds (COV - 69%; PID – 8%). For *LINC02541*, the average COV and PID across all vertebrates was 46% and 13%, respectively. There was some variation in the COV but not the PID which remained relatively consistent (COV - 66%; PID – 16%), mammals (COV - 38%: PID – 12%) and birds (COV - 69%; PID – 8%); although this variation is likely related to the smaller sample size.

Overall, these results suggest that both *MITA1 (IL7AS)* and *LINC02541* contain significant regions of conservation across vertebrates. To identify specific motifs/domains (up to 30 nucleotides), these sequences were submitted to the XSTREME option in MEME suite. This analysis identified 4 motifs/domains in *MITA1 (IL7AS)* and 13 motifs/domains in *LINC02541*, that were conserved across all the vertebrate species (**Figure 9B**).

### *MITA1 (IL7AS)* and *LINC02541* negatively regulate the LPS-induced inflammatory response in the human monocytic THP-1 cell line

**Figure 10:**
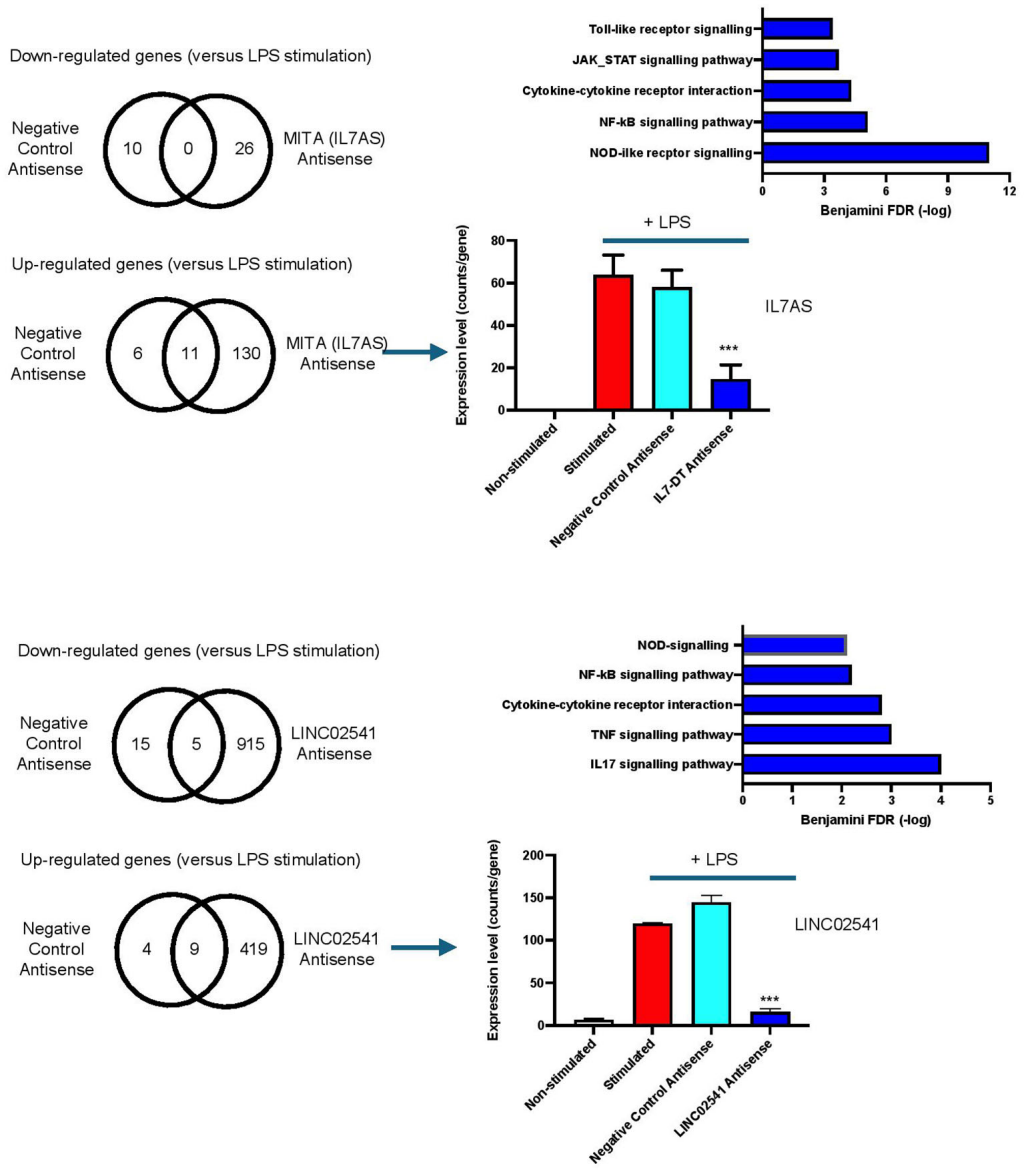
Knockdown studies demonstrate a function for *MITA (IL7AS)* and *LINC02541* expression in the LPS-induced inflammatory response in human THP-1 monocytic cells. Human monocytic THP-1 cells were transfected overnight with either an LNA-based antisense sequence against exon 1 in *MITA1 (IL7AS)* (MITA (IL7AS) Antisense), exon 3 in *LINC02541* (LINC02541 Antisense) or a scrambled negative control (Negative Control Antisense), stimulated for 4hr with LPS and then the change in the profile of mRNA expression determined by RNA sequencing. The number of up- and down-regulated mRNAs was determined following comparison with scrambled negative control before pathway analysis using DAVID (https://davidbioinformatics.nih.gov). Data is the mean +/- SEM of 3–4 experiments where ***p < 0.001 (unpaired T-test).

To determine the potential function of *MITA1 (IL7-AS)* and *LINC02541*, we designed LNA-based antisense sequences targeting exon 1 in *MITA1 (IL7AS)* and exon 3 in *LINC02541*, which are shared across the various predicted transcripts (**Supplementary Figure S1**). These were transfected into human monocytic THP-1 cells and their action upon the LPS-induced inflammatory response at 4hr was examined by RNA sequencing (**Figure 10**). Compared with a scrambled negative control, the targeting of exon 1 in *MITA1 (IL7AS)* (**Figure 10A**) resulted in the down-regulation of 26 mRNAs (including *MITA1 (IL7-AS)*) and the up-regulation of 130 mRNAs (**Figure 10A**; **Supplementary Table S43**). Pathway analysis indicated that the up-regulated genes were linked to multiple inflammatory pathways including the NF-κB and Toll-like receptor signaling pathways (**Figure 10A**). No pathways were associated with the down-regulated genes. Knockdown of exon 3 in *LINC02541* caused down-regulation of 915 mRNAs (including *LINC02541*) and up-regulation of 419 mRNAs, when compared with a scrambled control (**Figure 10B**; **Supplementary Table S44**). Once again, the down-regulated genes were not associated with any pathways whilst the up-regulated genes were linked with various inflammatory pathways including TNF and NF-κB signaling (**Figure 7B**). Overall, these studies indicate that *MITA1 (IL7AS)* and *LINC02541* are negative regulators of the LPS-induced inflammatory response and support the contention that syntenic conservation can provide an approach for the identification of functional lncRNAs in human phenotypic responses.

## Discussion

Comparative transcriptomics is a powerful technique for examining the differences in mRNA expression across species, as well as through evolutionary time. This approach is also central to investigating the role of long non-coding RNAs (lncRNAs), which can only be detected at the transcriptional level. However, the availability of data has commonly limited comparison between humans and other model organisms including mice and zebrafish. To address this limitation, we have mined all the transcriptomics data following LPS-induced activation of the innate immune response and created a unique resource containing data from 9 human cell types and 27 vertebrate species, including 7 primates, 10 mammals, 4 birds and 5 fish.

Initial comparison of the expression of genes involved in the LPS signaling pathway showed that this was highly conserved across vertebrates including primates (emerged ~70 mya), mammals (emerged ~120 mya), birds (emerged ~190 mya) and (jawed) fish (emerged ~420 mya), indicating that this has been present since the emergence of vertebrate (~480 mya). As previously reported, the only noticeable difference is the absence of the LPS sensing complex (TLR4/CD14/MD2) in fish (49), which appears to have evolved in vertebrates following their migration onto land (~ 480 mya). The observation that fish respond to LPS indicates that they must recognise this PAMP via an uncharacterised mechanism.

Having demonstrated conserved expression of the human LPS-signaling pathway across vertebrates, we proceeded to examine the LPS-induced transcription changes induced via this pathway. To this end, we compiled a high confidence list of LPS responsive mRNAs in humans by examining LPS-induced response in 9 different cell types. By selecting those that were differentially expressed in more than 4 cell types, we identified 1036 mRNAs that were employed for subsequent comparative transcriptomics. Based on orthology, we show evolutionary conservation between the LPS-induced mRNA changes in primates (82%), mammals (75%) and birds (75%), although this was dramatically reduced in fish (39%). Examination of individual genes that are induced across vertebrates identified many well characterised inflammatory modulators including IL-1β, CXCL8 and PTGS (COX2), as well as members of the NF-κB and AP1 transcription family. This also identified less characterised genes such as RNF19B, RIPK2, HBEGF, SDC4, NR4A3, PTX3 and BIRC3, that might warrant further investigation based on their evolutionary induction/conservation. Given the recent emergence of the importance of metabolism in the regulation of the immune response (50), it is also interesting to note the LPS-induction of multiple metabolic enzymes across vertebrates including SAT1, ACOD1, cytidine/uridine monophosphate kinase 2 and NAMPT.

Recent studies have highlighted the importance of lncRNAs in the regulation of the innate immune response (7–9). However, unlike protein-coding genes, their poor evolutionary conservation has precluded the search for lncRNAs based upon genomic DNA sequence. To address this challenge and identify functional lncRNAs that regulate human innate immunity, we have mined our datasets to identified conserved and differentially expression lncRNAs using transcriptional expression and syntenic position. This approach has previously been employed to compare tissue/cell specific lncRNA expression across vertebrates (10, 11). Our initial analysis in 9 human cell types identified 973 LPS-induced lncRNAs, of which 73 lncRNAs were differentially expressed in more than 4 cell types. Amongst this list of lncRNAs were the majority that had previously been shown to regulate the LPS-induced response in human cells including *MITA (IL7AS)* (8 cell types) (13, 51), *LINC02605* (7 cell types) (51), *ADORA2A-AS1* (52), *IL1β-RBT46* (1 cell type) (53), *PACER* (1 cell type) (54), *NEAT1* (3 cell types) (55) and *SUGCT-AS1* (3 cell types) (56), as well as the 4 lncRNAs recently identified by Schmerer et al. (19) including *ROCKI* (1 cell type) (57), *LINC00158* (6 cell types), *LINC01215* (2 cell types) and *AC022816.1* (6 cell types). Interestingly, there were a number that were expressed but not significantly up-regulated including *THRIL* (58), *MALAT1* (59), *HOTAIR* (60), *LINC00305* (61), *GAPLINC* (16), *lnc-IL7R* (62) and *CYP1B1-AS1* (63). This could be explained by alternative methods of detection (i.e. microarrays), cell type specific expression and/or the fact that their function is mediated by constitutive expression levels. In subsequent studies, we manually examined the syntenic conservation across 26 vertebrate species of the 73 lncRNAs that were induced in 4 or more human cell types. Remarkably, we found these lncRNAs were also syntenically expressed in primates (39 lncRNAs), mammals (32 lncRNAs), birds (5 lncRNAs) and fish (4 lncRNAs). Importantly, of these, many were shown to be significantly differentially expressed in response to LPS including 11 in primates and mammals and 2 in birds, with none in fish.

To assess whether syntenic conservation can identify human lncRNAs that regulate the LPS-induced innate immune response, we focused upon the two human lncRNAs whose syntenic versions are most commonly induced across vertebrate, *MITA1 (IL7AS)* (primates, mammals and birds) and *LINC02541* (primates and mammals).

*MITA1 (IL7AS)*, whose syntenic versions were induced in 18 vertebrate species, is located upstream of IL7, a hematopoietic growth factor that is important for lymphocyte development and multiple other immune functions (64). *MITA1* (metabolism-induced tumor activator 1) was named from studies in hepatocytes showing that its up-regulation is driven by energy stress and linked to hepatocellular carcinoma metastasis (65). Of relevance, this lncRNA was also identified in a screen for NF-κB dependent lncRNAs following TNFα- and LPS-stimulation of *p65^-/-^* and *Ikkβ^-/-^* mouse embryonic fibroblasts (MEF) and named *mNAIL* (mouse NF-κB Associated Immunoregulatory Long non-coding RNA) (66). In the current report, transcriptional profiling using RNAseq has shown that *MITA1 (IL7AS)* is a negative regulator of multiple inflammatory mediators including IL6, CCL2, CXCL8 and CXCL2 following LPS-stimulation of monocytic THP-1 cells. This is in agreement with our previous knockout studies examining the role of *MITA1 (IL7AS)* during IL6 release from monocytic THP-1 and mouse macrophage RAW264.7 cells (13). In contrast, studies in epithelial cells derived from the lung (A549) (13) and colon (SW480) (67) have shown that *MITA (IL7AS)* is a positive regulator of the release of IL6 and other inflammatory mediators. In the latter report, the regulation of CCL2, CCL5, CCL7 and IL6 expression by *MITA (IL7AS)* was mediated through an interaction with p300, that modulates the assembly of the SWI/SNF complex at their promoters (67). Significantly, *mNAIL* was also shown to positively regulate the release of inflammatory mediators *in vivo* using an LPS-induced model of endotoxemia, as well as a dextran sulfate sodium (DSS) induced model of colitis (66). These studies were performed using a mouse model *mNAIL*^ΔNF-κB^, in which the two NF-κB sites that drive *mNAIL* (e.g. *MITA (IL7AS)*) expression were removed. Mechanistic studies in the LPS-induced mouse model showed that *mNAIL* acts primarily in myeloid cells to promotes the release of inflammatory mediators through inhibition of Wip1 phosphatase, leading to activation of p38 MAP kinase and p65 transcriptional factors (66). Overall, these reports would indicate that the function and mechanism of action of *MITA1 (IL7AS)* is both cell- and/or species-dependent. This is agreement with previous observations that syntenic conservation does not always equate to functional and mechanistic conservation. Thus, *FAST* is a syntenically conserved lncRNA found in both mESCs and hESCs that differs in both its processing and localisation, that in turn impacts upon its function and mechanism of action (52).

In contrast to *MITA1 (IL7AS*), *LINC02541*, which was induced in 8 species, has not been previously implicated in the immune response. To ascertain the role of LINC02541, our knockdown studies showed that this lncRNA was also a negative regulator of the LPS-induced inflammatory response in human monocytic THP1 cells. To obtain additional information on *LINC002541*, we examined a number of lncRNA databases. In agreement with our transcriptional data, genomic data from LncBook 2.0 (68) identified homologs across 32 primate and mammalian species but not birds (chicken and zebrafinch) or fish (zebrafish). This database also identified SNPs that are associated with multiple carcinomas including haematopoietic and lymphoid carcinomas. Interestingly, a role in cancer is supported by publications showing that *LINC02541* promotes EMT in clear renal cell carcinoma (52) and following hypoxia (69). Mechanistic studies demonstrated that hypoxia/HIF-1α-driven *LINC02541* expression regulates core EMT regulators including TWIST1, SLUG and VEGF genes through an interaction with the YY1 complex and the promotion of histone 4 lysine 16 acetylation (H4K16Ac) marks (70, 71).

As with protein coding genes, it is currently believed that the actions of lncRNAs are mediated through domains that permits interactions with target proteins, RNA and/or DNA regions (4–6). However, the identification of these domains has been hindered by access to datasets on the lncRNA sequences across multiple species. Given that we have identified syntenic versions of *MITA1 (IL7AS)* in 18 species and *LINC02541* in 15 species, we extracted these sequences and compared them with the human version. Significantly, we have able to identify conserved regions and domains across these lncRNAs and speculate that these might be important in mediating their negative regulation of the innate immune response.

In summary, the LPS-induced response is unique in the fact that we have transcriptional data spanning 27 vertebrate species ranging through humans, primates, mammals, birds and fish. Our annotation and compilation of this data will provide an important resource for the future analysis of individual mRNAs and lncRNAs both across vertebrate species and in the context of evolution. Specifically, we believe that the identification of syntenically conserved lncRNAs will aid in the identification of functional lncRNAs in the human innate immune response, as well as the elucidation of their mechanism of action.

## Data Availability

The datasets for this study can be found at the GEO SRA using the accession numbers listed in **Figure 1** and **2**.
